# Regulators of Starch Biosynthesis in Cereal Crops

**DOI:** 10.3390/molecules26237092

**Published:** 2021-11-24

**Authors:** Ruiqing Li, Yuanyuan Tan, Huali Zhang

**Affiliations:** 1 State Key Laboratory of Rice Biology, Chinese National Center for Rice Improvement, China National Rice Research Institute, Hangzhou 310029, China; liruiqing@ahau.edu.cn; 2 College of Agronomy, Anhui Agricultural University, Hefei 230036, China; 3 National Key Laboratory of Rice Biology, Institute of Crop Sciences, College of Agriculture and Biotechnology, Zhejiang University, Hangzhou 310029, China; tanyy@zju.edu.cn

**Keywords:** starch biosynthesis, cereal crops, transcription factors, regulator, endosperm

## Abstract

Starch is the main food source for human beings and livestock all over the world, and it is also the raw material for production of industrial alcohol and biofuel. A considerable part of the world’s annual starch production comes from crops and their seeds. With the increasing demand for starch from food and non-food industries and the growing loss of arable land due to urbanization, understanding starch biosynthesis and its regulators is essential to produce the desirable traits as well as more and better polymers via biotechnological approaches in cereal crops. Because of the complexity and flexibility of carbon allocation in the formation of endosperm starch, cereal crops require a broad range of enzymes and one matching network of regulators to control the providential functioning of these starch biosynthetic enzymes. Here, we comprehensively summarize the current knowledge about regulatory factors of starch biosynthesis in cereal crops, with an emphasis on the transcription factors that directly regulate starch biosynthesis. This review will provide new insights for the manipulation of bioengineering and starch biosynthesis to improve starch yields or qualities in our diets and in industry.

## 1. Introduction

As a fundamental commodity, starch was and is still widely used for human consumption in food and non-food industries [1,2]. Since starch is the major storage carbohydrate and generally accumulated in the heterotrophic starch-storing organs of crops [3,4,5], a large amount of starch is sourced from the attainable parts of staple crop plants [5], especially for cereal seeds, roots, and tubers. Among them, cereal seeds make up most of the worldwide annual starch production.

Starch is composed of amylose and amylopectin glucan polymers, which are packaged to form the insoluble semi-crystalline starch granules [6]. Starch synthesized in cereal crops contains at least two types, termed transitory starch and storage starch. Transitory starch is usually found in the plastid of photosynthetic organs and displays circadian turnover regulation with diurnal cycles [7]. While the storage starch is synthesized in the amyloplasts of non-photosynthetic sink tissues (i.e., seed endosperm), and requires the supply of sucrose and ATP from the source organs (i.e., leaves) [5]. Interestingly, some common regulatory factors of starch synthesis exist between chloroplasts and amyloplasts. For example, the expression of starch biosynthetic genes, i.e., *GBSSI*, was partly mediated by tetrapyrrole intermediates, i.e., heme, in endosperm during early seed development in rice [8], suggesting potential shared regulatory networks in various plastids.

The process of starch biosynthesis in crops requires tight cooperation of various starch biosynthetic enzymes, and coordinates with other metabolisms. Until now, a great number of genes involved in starch biosynthesis have been identified in various cereals. However, despite these findings, the regulatory factors underlying starch synthesis are still poorly understood, especially regarding the regulation of starch biosynthetic genes. Therefore, the screening and identification of key regulators is vital to understanding the fundamental regulatory mechanism underlying starch synthesis. Here, we comprehensively provide an update on current knowledge on the pivotal spatiotemporal-dependent regulators of starch biosynthesis in cereal crops with a focus on transcription factors directly regulating starch biosynthesis, which will provide new insights into cereal starch yield or quality improvement on our diet as well as in industry.

## 2. Transcription Factors Directly Regulating Starch Biosynthesis

**Figure 1:**
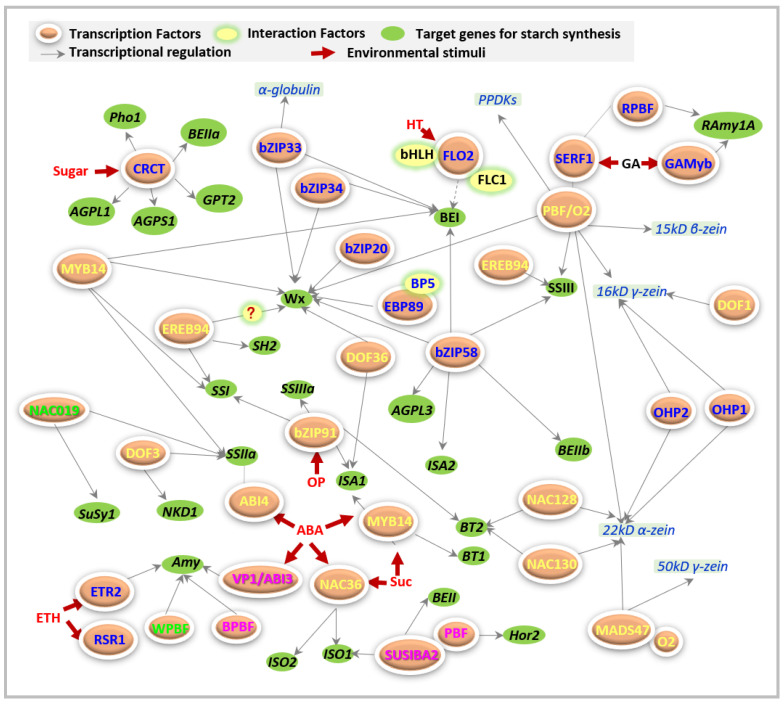
Overview of the regulators of starch metabolism in cereal crops. The figure was created based on the published literature (until 28 August 2021). The reported regulators and their target genes from different cereals were collected to build potential regulatory networks of starch metabolic genes. Starch biosynthesis involves a great deal of enzymatic and non-enzymatic proteins, which constitutes a very complex network. This process requires many regulators to match the providential functioning of these starch biosynthetic enzymes. Nowadays, dozens of transcription factors have been reported to directly regulate starch synthesis, i.e., bZIP58, NAC019, MYB14, FLO2, SUSIBA2, and so on. Besides, some regulators also control starch synthesis through the formation of proteins and sucrose, including MADS47, DOF1, and so on. Various regulators prefer to be activated in response to different fluctuating environments, such as hormone levels (i.e., ethylene, ETH; abscisic acid, ABA; gibberellin, GA), osmotic pressure (OP), sugar (i.e., sucrose, SUC), and high temperature (HT), etc. Regulators that exist in rice, maize, wheat, and barley are shown in blue, yellow, purple, and green color letters, respectively.

Starch biosynthesis is a complex process and requires various functional enzymes, which are regulated by transcription factors (TFs) and/or other regulators (Figure 1). Transcription factors mediate gene expression through direct combination with various domains in the promoter regions of target genes based on different environmental factors [9]. Likewise, regulation of the starch synthetic genes is also subjected to the regulation of many TF families (Figure 1), including basic leucine zipper transcription (bZIP) [10,11], APETALA2/Ethylene-Responsive Factor (AP2/ERF) [12,13,14], NAC (no apical meristem (NAM), ATAF1/2, cup-shaped cotyledon (CUC2)) [15], MYB [16], GRAS [17], and DNA binding with one finger (DOF) [18,19].

Transcriptional regulation of starch biosynthesis varies with the environment, such as phytohormones and sucrose, and shows spatial-temporal gene targeting in different cereal crops. Based on the expression patterns in various tissues, genes involved in starch synthesis can be divided into two types [14,20]. The type I genes are more likely to be expressed in sink organs (e.g., endosperm), while the type II genes are abundantly expressed in source organs, including the root, seedling, leaf, ovary, and embryo [14,20]. Accordingly, the TF members of the bZIP, AP2/ERF, MYB, or NAC families always co-express with type I, whereas the bHLH (for basic helix-loop-helix), homeobox, or SET (Su(var)3-9, Enhancer-of-zeste, and Trithorax) families mostly co-express with type II [14]. These findings indicate that TF-involved starch biosynthesis has somewhat tissue-specific characteristics.

### 2.1. bZIP

bZIP factors are the first identified transcription factors to regulate starch biosynthesis [21,22], and they appear to be one of the main regulators of this process. bZIP regulators have been well characterized in several crops, including *Oryza sativa* L. [23,24], *Zea mays* L. [10], *Hordeum vulgare* L. [25], and *Triticum aestivum* L. [26,27]. bZIP factors play multiple roles during starch synthesis, but their precise roles vary in different species. This divergence includes a set of target genes and the binding affinity. For example, one bZIP transcription factor, OsbZIP58, regulates six genes during starch metabolism in rice [10,28,29], while the identified candidate TabZIPs are only involved in the regulation of granule-bound starch synthase I (GBSSI)/Waxy (Wx) and branching enzyme II (BEII) during starch biosynthesis in wheat [26]. Furthermore, the binding motif of bZIPs seems to be considerably the same in rice [11], barley [25], and maize [23], but it is still not clear whether a similar phenomenon exists in other cereal species.

**Figure 2:**
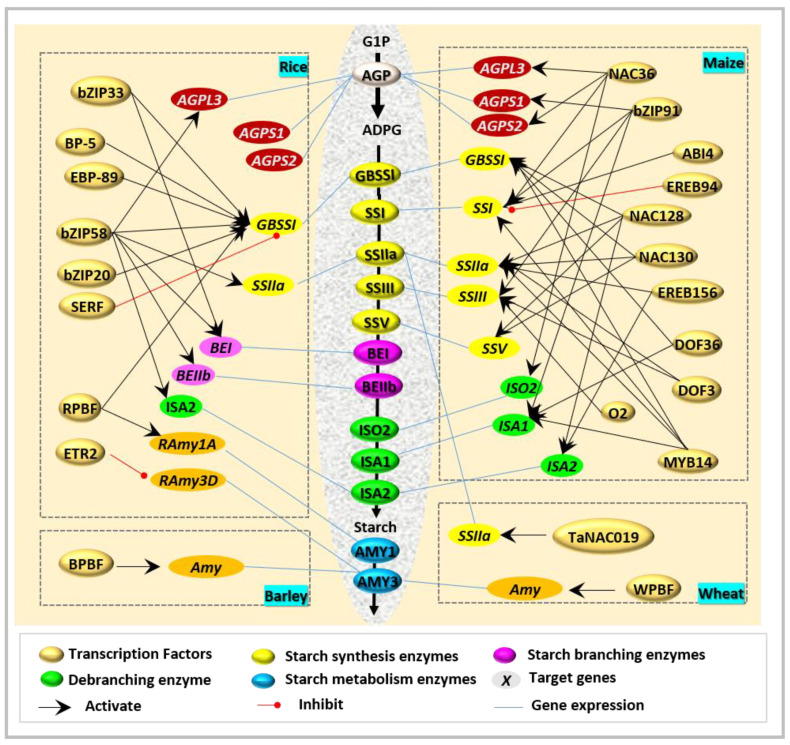
TF-regulating genes that encode starch biosynthetic enzymes. The formation of starch in cereal crops is the result of a series of enzymatic reactions, which require the cooperation of many TFs. Regulation of TFs on starch biosynthetic genes showed the following patterns: (i) the same gene was regulated by several TFs, i.e., *GBSSI* or *Wx* to *RPBF*, *bZIP58*, *BP-5*, and *EBP-89*, etc.; (ii) several genes were regulated by the same TF, i.e., ZmNAC128 and ZmNAC130; (iii) the regulation of TFs was also affected by various environments. Besides, there were regulatory differences of TFs in different cereals. For example, OsbZIP58 was close to ZmbZIP91 and O2, but their target genes were divergent.

bZIPs show broad affinities with different motifs in the process of regulating starch biosynthesis. A well-studied bZIP in rice, OsbZIP58 (also called RISBZ1 or OsSMF1) [28,29], has been revealed as a regulator of starch biosynthetic genes [11]. OsbZIP58 interacts with different motifs to regulate starch biosynthesis (Figure 2 and Table 1), and mainly functions through three manners, including (i) by binding to the ACGT (CCACGTG/C) element to regulate the synthesis of storage proteins and free lysine [18,30,31]; (ii) by direct combination with the motif of ACGT to regulate the expression of *ADP-glucose pyrophosphorylase large subunit 3* (*OsAGPL3*), *OsGBSSI*/*Wx*, *starch synthase IIa* (*OsSSIIa*), *BEI*, *OsBEIIb*, and *Isoamylase-type starch debranching enzyme 2* (*ISA2*) [11]; and (iii) by activating the synthesis of several seed storage proteins and the expression of two NAC transcription factors associated with starch biosynthesis [29,30] by binding with the GCN4 or ACGT motif. Moreover, several other bZIPs also exhibit broad affinity to the GCN4 or ACGT motif [29] upon their divergent functions. OsbZIP20 (RITA, rice transcription activator-1) interacts with the palindromic ACGT elements to mediate starch synthesis [24], whereas OsbZIP33 (rice endosperm basic leucine zipper, REB) shows binding specificity with ACGT elements in the promoters of *BEI* and *GBSSI* during starch synthesis [32]. Similar phenomena also exist in maize. Maize Opaque2 (O2) [10] could directly regulate *starch synthase III* (*SSIII*) and indirectly regulate *SSIIa* and *BEI* via interaction with prolamin-box-binding factor (PBF) to mediate starch biosynthesis at transcriptional levels. Moreover, ZmbZIP91 [33] binds to the ACTCAT elements to regulate starch synthesis genes (*pAGPS1*, *pSSI*, *pSSIIIa*, and *pISA1*).

Besides, the functions of bZIPs depend on tissue types to some extent. OsZIP58 shows highly endosperm-dependent expression during seed development [11,28], but its highly orthologous genes (e.g., ZmbZIP60, ZmbZIP16, ZmbZIP17, ZmbZIP91) [33] are widely expressed in all types of tissues in maize. Simultaneously, TabZIP [26] and one TubZIP28 and its homolog from *Triticum aestivum*, TabZIP28 [27], were also reported to positively regulate starch biosynthesis (Table 1).

Interestingly, ZmbZIP91 [33] and O2 [10] are the two closest homologous bZIP transcription factors of OsbZIP58. However, their function seems not to be fully in line with OsZIP58, and has divergent target genes and tissue dependence. ZmbZIP91 could bind to the ACTCAT directly to regulate the expression of *AGPS1*, *SSI*, *SSIIIa*, and *ISA1* during starch synthesis [33], while O2 [10] usually combines with other factors in the regulation of starch biosynthesis (Figure 2).

In addition to the heterodimers of bZIPs (e.g., O2) and DOF family factors (e.g., PBF), the MYC proteins also often interact with the ethylene-responsive element-binding protein (EREBP) family in their regulatory functions during starch biosynthesis. For example, one MYC-like protein, OsBP-5, has been reported to interact with the OsEBP-89 (*Oryza sativa* EREBP clone 89) protein to regulate the *Wx* gene expression in rice [12]. The binding motifs of rice-binding protein 5 (OsBP-5; CAACGTG) is in close proximity to OsEBP-89 (GCCAAC) in the *Wx* promoter [12]. However, the precise affinity and specificity of different binding motifs remain to be further studied.

### 2.2. AP2/ERF

AP2/ERFs serve as one key regulator in the ethylene- and its receptor-mediated signals [16], and is also involved in starch metabolism and accumulation [34,46]. Nowadays, the AP2/ERF family includes three individual subfamilies (ERF, AP2, and RAV) [39]. Among them, ERF factors have been well demonstrated to regulate starch biosynthesis in cereal crops, e.g., *Oryza sativa* L. [14,34], *Zea mays* L. [37,39], *Hordeum vulgare* L. [36], and *Triticum aestivum* L. [13]. In general, the AP2/ERF factors can combine with DRE, GCC, or CAACA box [47] in the promoter regions of target starch synthetic genes.

AP2/ERF factors probably function through the coordination between different biological processes. One AP2/EREBP-type regulator, Rice Starch Regulator1 (RSR1), has been reported to negatively regulate starch synthesis-associated genes both in wheat [13] and rice [14], but its regulatory mechanism is still unknown. Moreover, OsRSR1 is not only co-expressed with starch synthesis but also co-expressed with most of the genes involved in photosynthesis in leaves. One possible explanation is that starch is the primary product of photosynthesis, which is regulated through chloroplast formation and chlorophyll biosynthesis [14]. Other evidence is from ZmABI4 (ABA Insensitive 4) in maize. As an AP2/ERF family member, ZmABI4 had negative effects on the transcriptional abundance of photosynthesis-associated nuclear genes [48]. Therefore, the tight association between photosynthesis and starch biosynthesis is required for the precise regulatory mechanisms in response to various tissues and environments.

Multiple AP2/ERF factors usually regulate the starch synthesis genes through the induction of phytohormones, including ethylene, abscisic acid (ABA), and gibberellin (GA; Figure 1 and Table 1). Two subfamily II ethylene receptors, ETR2 and *Sub*1C, are reported to promote starch accumulation by depressing the *α*-amylase gene *RAmy3D* in rice internodes [34] and leaves [38]. These processes are in response to ethylene-dependent developmental stages, employing a common signaling pathway of OsETR2-OsGI [34].

ABA-induced AP2/ERF in starch synthesis includes at least two TFs, EREB and ABI (ABA insensitive; Figure 2). Starch synthesis is positively associated with the ABA level in rice [49], wheat [50], and barley [51]. ABA plus sucrose-induced ZmEREB156 could directly bind to the promoter of *ZmSSIIIa* to positively mediate starch synthesis [52]. On the contrary, ZmEREB94 has negative effects on starch synthesis through direct combination with the *ZmSSI* promoter and indirect regulation of *ZmSh2* and *ZmGBSSI* during seed development [39]. The ABA-mediated signaling also depends on three ABI transcription factors (ABI3-ABI5) during seed development [36,37,53,54]. ABI3 [36] and ABI4 [37] regulate starch synthesis via mediation of the expression of *GAMYB* and *SSI* genes, respectively. However, ABI3, also named viviparous-1 (VP1), was previously characterized as one regulator of seed development in maize [55,56] and then found to command different spatiotemporal expression pattern specificities in barley [36]. HvABI3/VP1 mediates the GAMYB/BPBF-activated *Hor2* (For B-hordeins) expression in developing endosperm and activates the GAMYB-mediated *Amy6.4* expression in post-germinative reserve mobilization [36]. The ABA-induced ABI4 also interacts with a CACCG box in the promoter of *ZmSSI* [37] to mediate starch synthesis. Besides, many other binding locations, including the coupling element1 (CE1)-like motif (CACCK) [57], the S box (CACYKSCA) [48], and the CCAC motif [58,59], are also involved in the regulation of gene expression induced by sugar or ABA. Thus, ABI4 might function with different motifs through various signaling pathways, depending on the divergence from tissues, organs, and species, as well as the developmental stages.

Several AP2/ERF TFs are also induced by GA in starch synthesis, i.e., MYB and SALT-RESPONSIVE ERF1 (SERF1; Figure 2). GAMYB is a gibberellin-induced transcription factor that activates seed storage genes and hydrolase genes by recognizing the GARE motif [44,60,61]. Another gibberellin-mediated AP2 family TF, rice SERF1, also participated in the regulation of starch biosynthesis [35], and negatively mediated the accumulation and remobilization of starch through the direct regulation of *RPBF* and *GBSSI* in the presence of GA [35]. RPBF is one of the P-box binding DOFs [25], and thereby, we will elaborate on its functions in the section of “DOF (DNA binding with one finger) proteins”.

Obviously, phytohormone-induced transcriptional regulation requires cooperation with a different set of TFs in accord with their balance. This is also found in other cereal species. For example, OsEBP89 can form a heterodimer with one MYC transcriptional factor, OsBP-5, to regulate *GBSSI* expression synergistically in rice, and the GCCAAC binding box of OsEBP89 is next to the CAACGTG binding motif of OsBP-5 in the promoter of *GBSSI* [12]. Besides, as a key enzyme for starch synthesis, GBSSI has several different motifs (e.g., P-box and GCN4) in its regulatory regions, so multiple TFs, including RISBZ1 (for GCN4 motif binding) [11], OsBP-5, and OsEBP-89 [12], could directly bind and trans-activate *GBSSI* expression. Besides, PBF can also bind to P-box located in the promoter of *GBSSI*, and thus, *GBSSI* expression can also be indirectly regulated by the direct binding of SERF1 to PBF [35]. Thus, the functions of the regulators during starch biosynthesis show spatiotemporal expression pattern specificities and require cooperation of multiple transcriptional factors.

### 2.3. NAC

Transcription factors of NAC (No Apical Meristem (NAM), ATAF1/2, cup-shaped cotyledon (CUC2)) represent one of the largest families in plants and are involved in the stress-induced response during development periods [29,62]. Yet, NACs of starch biosynthesis in cereal crops have relative conservation. For example, the maize NAC36 factor was identified based on the sequence homology of one reported rice gene [15]. However, the regulatory functions of NACs on starch synthesis have remained unclear until the recent reports of two TFs, ZmNAC128 and ZmNAC130.

ZmNAC128 and ZmNAC130 are specifically expressed in grain endosperm during the filling stage, and thus function in a spatiotemporal pattern [40]. These two NACs are not only regulators involved in starch synthesis, but also underly the regulatory mechanism of the synchronization of utilization on carbon metabolism [40]. ZmNAC128 and ZmNAC130 mediate starch synthesis in the endosperm [40] through binding to the ACGCAA motif in the promoters of *Brittle 2* (*BT2*) and six other hypothetic genes, including *Zpu1* (encoding *Zea mays pullulanase-type starch debranching enzyme*), GBSSI, *Sh2* (for *AGPase large subunit*), *SSV*, *ISA2*, and *SSIIa* (Figure 2 and Table 1). In addition, two related NACs were identified by using the blasting of protein sequences in rice, which show the same phenomenon of selective pressure [40]. More importantly, syntenic alignments among divergent cereal genomes of some TFs have been shown throughout cereal evolution [63], but sequence similarity is not the whole story. For example, rice OsZIP58 [28] displays a similar function to maize O2 in the same mode [11], but its other highly orthologous (e.g., ZmbZIP60, ZmbZIP16, ZmbZIP17, ZmbZIP91) show great expression divergences in maize [33].

Recently, a novel NAC-type transcription factor was revealed to regulate starch biosynthesis in wheat. TaNAC019 is specifically expressed in endosperm, and functions during starch synthesis through the following ways [41]: (i) by direct binding to the protomers of glutenin-encoded genes, (ii) by mediation of the accumulation of storage proteins via regulated *Wheat storage protein activator* (*TaSPA*) expression and interaction with TaGAMyb, and (iii) by adjustment of the accumulation of starch by the regulation of SSIIa and Susy1.

### 2.4. MYB

As the widely distributed transcription factors, MYBs are vital for a variety of biological functions. However, the functional studies of MYBs on starch biosynthesis are still scarce in cereal crops. So far, only one MYB factor has been reported as a regulator during starch biosynthesis [16].

Although many MYBs, i.e., ZmMYB73, ZmMYB127, ZmMYB155, and ZmMYB14, seemed to play roles in starch biosynthesis, only ZmMYB14 [16] has been evidently identified to promote endosperm starch synthetic genes in maize (Figure 2). Besides, there are two points of contention remaining over ZmMYB14. One is that the binding motif of *ZmBT1* for ZmMYB14 to function as a regulator of starch synthesis may not be through the MBSI site (TAACTG) but other sites or mechanisms, while another is that there are spatial-temporal differences between type I starch synthesis-associated genes and ZmMYB14 [16], which might be due to programmed cell death in endosperm. So, MYBs could function as important regulators related to starch biosynthesis, but more studies are required to reveal and decrypt the mechanisms of MYB factors during starch biosynthesis in cereal crops.

### 2.5. DOF

DOFs show endosperm-specific expression in the regulation of starch biosynthesis (Figure 2 and Table 1). Regulation of DOFs on starch biosynthesis is mainly reflected in seed development and germination [14]. Prolamin-box-binding factor (PBF) is one of the major endosperm-specific DOF family members. Nowadays, increasingly more cereal PBFs have been identified, including rice PBF (RPBF) [18], maize PBF (PBF) [10], barley PBF (BPBF) [18,44], and wheat PBF (WPBF) [45]. However, PBF usually performs regulatory roles in starch biosynthesis combined with other regulator factors, i.e., O2 [10], SERF [18], and MYB [36]. Nonetheless, there are diverse mechanisms that exist between seed development and seed germination. During rice grain development, the expression of *RPBF* was negatively regulated by SERF1, displaying contrasting expression patterns [18,35], while during seed germination, the GAMyb-induced expression of *RAmy1A* was enhanced in the presence of RPBF [64,65]. One similar phenomenon has also been reported in *Hordeum vulgare*. GAMYB and BPBF in the developing endosperm activates the full *Hor2* expression controlled by the expression pattern of HvVP1 (VIVIPAROUS1), orthologous to ABI3 from *Arabidopsis thaliana*, whereas the activation of Amy6.4 in post-germinative reserve mobilization was mediated by GAMYB [36]. So, it seems that these processes share the regulation of AMY activity. Moreover, O2/PBF mediates the enzyme complex via the transactivation of PPDKs and SSIII during starch biosynthesis [10].

DOFs also mediated the synthesis of starch and seed storage proteins (SSPs) through recognition of the AAAG motif [66]. Several DOFs function as positive regulators to mediate starch accumulation during endosperm development. For example, three DOFs have been identified to regulate starch synthesis in maize, including ZmDOF1 [67], ZmDOF3 [16], and ZmDOF36 [43], which have different target genes (Figure 2). ZmDOF36 regulates *ZmGBSSI* and *ZmISA1* through the combination with DOF core elements in their promoters both in vitro and in vivo [43], while ZmDOF3 could interact with the DOF core element of *SSIIa* [16].

### 2.6. GRAS

GRAS transcription factors are involved in plant growth and development processes, especially for endodermis specification [68]. Besides, GRAS is greatly associated with type I genes during endosperm starch synthesis in rice [14], but its function as a regulator for starch biosynthesis is still not understood in cereal crops.

One maize TF of GRAS, ZmGRAS20, plays regulatory roles in starch biosynthesis of rice endosperm [17]. Through transcriptome sequencing, ZmGRAS20 was selected and showed high expression both in developing maize grains [69] and in ZmGRAS20-overexpressing rice seeds [17], but its specific expression parts were endosperm. Besides, ZmGRAS20 overexpression in rice leads to a chalkiness characteristic with an altered starch content and structure [17]. The molecular roles of GARS20 in starch synthesis require further studies.

### 2.7. WRKY

Sugar signaling in barley2 (SUSIBA2) is a plant-specific WRKY TF that regulates gene expression in the presence of sugar, thereby mediating the communication of source-sink [42,70] via a cis element of SURE (sugar responsive) in plant sugar signaling. SURE was first reported in potato [71], and was subsequently isolated from rice, barley, and wheat [70]. SUSIBA2 serves as a regulatory transcriptional factor to regulate sugar-responsive gene expression, i.e., *isoamylase* (*ISO*), and thereby mediates carbohydrate anabolism [70]. Interestingly, the heterologous expression of *SUSIBA2* produced a rice variety with a high starch content but low methane emission [72], suggesting its important function in starch biosynthesis. In parallel, by using the antisense oligodeoxynucleotide (ODN) technology, one SUSIBA2 regulatory TF has been identified in barley [42]. SUSIBA2 binds to the SURE elements in the promoters of *ISO1* and *BEIIb*, and confers tissue-specific sugar responsiveness [42,70]. Therefore, as a WRKY regulatory TF of sugar signaling, SUSIBA2 possesses some specific characteristics in cereals, and regulates starch biosynthesis through starch synthesis genes, i.e., *ISO* and *BE*.

## 3. Other Regulators Directly Affecting Starch Biosynthesis

Some regulatory factors cannot be listed in any of the above groups, and thus are included independently in this section (Figure 1), including FLOURY ENDOSPERM (FLO), CO_2_-responsive CONSTANS, CONSTANS-like, and time of chlorophyll a/b-binding protein1 (CRCT).

### 3.1. Floury Endosperm

*FLOURY ENDOSPREM* is named based on its well-recognized but viable endosperm phenotypes, which usually display a chalky and soft endosperm. So far, 16 rice floury mutants [73] and three maize floury mutants [74] have been identified. However, only rice FLO2 [75] and FLO7 [76], as well as *ZmFloury3* [74] serve as regulators of starch biosynthesis but do not act as enzymes. Although all of them play key roles in the regulation of starch formation, and are also responsible for aberrant seeds with reduced grain quality [74,75,76], their functioning models vary.

FLO2 acts as a regulatory protein that controls the biosynthesis of seed storage substances, possibly through its tetratricopeptide repeat (TPR) motifs for protein–protein interactions [75]. At least two proteins have been reported that interact with FLO2 during starch synthesis (Figure 1). One basic helix–loop–helix (bHLH) protein may interact with FLO2 to modulate the expression of starch biosynthesis-associated nuclear genes (SBANGs) [75]. Subsequently, the FLO2-interacting cupin domain protein 1 (FLOC1) was reported to interact with the TPR motif of FLO2 to maintain fertility and seed quality in rice [77], even though they are supposed to have different functions.

FLO7 prefers to specifically function in the endosperm periphery, and harbors two essential domains of an *N*-terminal transit peptide and an unknown function 1338 (DUF1338), during starch synthesis and amyloplast development [76].

Floury3 (named FL3), a plant AT-rich sequence and zinc-binding (PLATZ) protein, acts as a specific regulator in maize starchy endosperm cells [74]. FL3 plays vital roles in endosperms during seed-filling periods through interactions with RNA polymerase III subunit 53 (RPC53) and transcription factor class C 1 (TFC1), which are two critical factors of the RNA polymerase III (RNAPIII) transcription complex [74]. Interestingly, FL3 was regulated by genomic imprinting [74], and thus, its mutation caused a semidominant-negative mutant.

### 3.2. CRCT

The CRCT protein has been reported to be a regulator of starch biosynthesis in rice but does not seem to belong to a zinc finger motif [78], and cannot be listed in any of the above groups. Thus, here, CRCT is grouped independently. The expression of *OsCRCT* displays diurnal oscillation and is mainly focused on the vegetative organs. Therefore, OsCRCT serves as a positive regulator of several starch biosynthetic genes, including *AGPS1*, *AGPL1*, *Glc-6-phosphate translocator2* (*GPT2*), *plastidial α-glucan phosphorylase1* (*Pho1*), and *BEIIa*, and contributes to starch biosynthesis in rice leaves [78]. The effects of OsCRCT on the storage of starch are unclear so far. However, the use of plants overexpressing *OsCRCT* is a potential approach to improve yields for food and biofuel [78], depending on the increased contents of starch derived from enhanced synthesis of starch instead of the defects of starch breakdown or translocation.

## 4. Regulators Indirectly Mediating Starch Biosynthesis

Aside from the direct regulators of starch biosynthesis, many other factors also participate in the regulation of starch synthesis, partially through the post-transcriptional regulation of TFs, i.e., micro RNAs (miRNAs), or via regulators of the formation of the upstream precursors and/or downstream products of starch.

### 4.1. miRNAs

miRNAs can negatively mediate gene expression at the post-transcription levels [79], and have regulatory roles in plant development, e.g., hormone signaling, stress responses, and so on [80,81]. miRNAs have been reported to regulate starch accumulation during grain filling in cereal crops, including rice [82], wheat [83], and maize [84,85]. In rice, miR167 regulates seed formation through a confirmed pathway of auxin-miR167-ARF8-OsGH3.2 (encoding Indole-3-acetic acid-amido synthetase) [18,82]. Yet, OsGH3.2 seems to have no direct correlation with starch biosynthesis, but the target mRNAs of many sucrose-induced miRNAs, i.e., miR159, miR397, miR160, miR529, miR166, miR528, miR167, miR171, miR398, and miR827, are characterized as transcription factors [84], including AP2/ERF, ARF, GRAS, MYB, NAC, and WRKY in maize. Most of these TFs are involved in endosperm starch biosynthesis, suggesting that the sucrose-induced regulation of starch synthetic genes is possibly through miRNAs. However, the related signaling pathway among them remains largely unknown.

### 4.2. TFs Involved in the Regulation of Sugars and Proteins

Some transcription factors have been reported to mediate starch accumulation in cereal endosperms, but they participate in regulation via control of the synthesis of the downstream and/or upstream products of starch, i.e., sugar and proteins, instead of directly regulating starch synthesis. Here, we mainly concentrate on some of them (Table 2), including bZIPs, NAC, DOF, MADS, and basic helix-loop-helix (bHLH).

#### 4.2.1. bZIP

Aside from direct regulation of starch synthetic enzymes, some bZIPs are also involved in the regulation of starch synthesis via indirect ways (Table 2). For example, O2 can regulate the gene expression of *α*- and *β*-zein [23], *b-32* [21], and *pyruvate orthophosphate dikinase 1* (*PPDK1*) [10,22] via recognition of the O2 box, GA/TGAPyPuTGPu, and TCCACGTAGA sequences in their promoters, respectively. Moreover, two heterodimerizing proteins of O2, OHP1 and OHP2, can regulate the synthesis of 27-kDa *γ*-zein and 22-kDa *α*-zein [86,87] through the recognition of the O2-like motif in the promoters of the target genes. O2, PBF, and OHP1/OHP2 can jointly form a complex with ZmbZIP22 [86] to combine with the ACAGCTCA motif in the promoter of γ-zein (27-kDa) [87]. Besides, the interaction of O2 with ZmMADS47, an MADS box-containing TF [89], is essential to activate *α*-zein and 50-kDa *γ*-zein through the binding of the CATGT motif in their promoters.

#### 4.2.2. AP2/ERF

Recently, one B3 domain-containing AP2/ERF transcriptional factor, ZmABI19 [88], was well documented to mediate starch synthesis via binding with RY through the regulation of cooperated expression of *O2*, *PBF1*, *ZmbZIP22*, *NAC130*, and *O11*.

#### 4.2.3. NAC

Regulation of NAC on starch synthetic enzymes is often accompanied with a simultaneous regulation of storage protein synthesis-associated genes (Table 2). Although ZmNAC128 and ZmNAC130 have been well documented to regulate several starch biosynthetic genes, they can also bind with the motif of ACGCAA to activate the expression of *16-kDa γ-zein* genes [40]. However, these two NAC factors [40] do not dimerize with each other but are functionally redundant for starch and protein accumulation. A similar phenomenon was also found in wheat [41]. TaNAC019 [41] has been reported to directly bind to the promoters of glutenin-encoded genes and be involved in the accumulation of starch and proteins. Therefore, the regulation of starch biosynthesis is not independent but prefers to act synergistically with other metabolic processes, i.e., protein synthesis.

#### 4.2.4. DOF

Some DOF TFs have similar effects on the regulation of starch synthesis. PBF specifically binds with P-box (TGTAAAG) to mediate the synthesis of proteins (Table 2), i.e., *α*-zein and *γ*-zein [64]. PBF can also interact with O2, PBF, and OHP1/OHP2 to form a complex with ZmbZIP22 [86] to regulate the expression of *27-kDa γ-zein* [85]. Therefore, the correlation of PBF and other factors is greatly related to seed size, vigor, and germination. Moreover, other DOFs mediate seed storage proteins (SSPs) through recognition of the AAAG motif [67] during seed development. DOF1 promotes the upregulated expression of *γ-zein* [64], while ZmDOF3 [16] can bind to the *Nkd1* (*naked endosperm 1*) promoter to regulate aleurone cell differentiation.

#### 4.2.5. MADS

MADS-box transcription factors have attracted increasingly more attention in the studies of seed development [68,90]. For example, ZmMADS1, a typical MADS-box transcription factor, regulates sugar synthesis-associated genes, and functions to affect the endosperm characteristics of maize seeds as well as in resistance to drought stress [45]. A similar phenomenon also exists in rice endosperm [45,90]. This is largely dependent on the similar adjustment mechanism of ZmMADS1, which shows enhanced expression of starch biosynthetic genes, i.e., *GBSSI*, in mature seeds. Another MADS TF, ZmMADS47, through the interactions with OPAQUE2, directly regulates prolamin in maize [89]. Interestingly, in rice, OsMADS29 has been explored to produce shrunken seeds, but the underlying mechanism prefers programmed cell deaths (PCDs) [91]. However, ZmES22, one of the MADS TFs, has been documented to negatively regulate endosperm starch accumulation in rice via direct downregulation of the expression of *OsGIF1* during grain filling stages [92]. Thus, the regulation of MADS TFs on starch biosynthesis depends on various ways, i.e., (i) by adjusting sugars, (ii) by mediating prolamins, (iii) and through PCDs, and so on.

#### 4.2.6. bHLH

TFs of basic helix–loop–helix act as important regulators of starch synthesis during seed formation [93,94]. For example, maize opaque11 showed endosperm-specific expression and participated in the regulation of *NKD2* and *ZmDOF3* during endosperm development. Besides, it is also involved in the regulation of nutrient and carbohydrate metabolism via O2 and PBF, as well as in response to environmental stresses. Thus, OPAQUE11 may work as the focus hub in the regulatory network during seed formation [94].

ZHOUP1 (ZOU) is involved in the seed formation of angiosperm plants, i.e., *Arabidopsis thaliana* [95,96] and maize [93]. ZmZOU is specifically expressed in endosperm, especially during the filling periods [94]. Similar interaction mechanisms for seed development are also found in maize [93] and in *Arabidopsis* [96,97], via the formation of functional heterodimers. However, it is essential for ZmZOU to tightly bind with its specific ZmICEa in the formation of grain filling. Thus, this seems to be unique for monocotyledon plants. All in all, ZHOUP1 does not directly affect starch biosynthesis but plays vital roles in the formation of endosperm and embryo.

## 5. Conclusions

Starch was and remains an essential commodity in food and non-food industries. Cereal seeds provide a great account of starch production, and thereby, starch synthesis and its regulation in seed endosperm have been investigated by researchers of breeding. The enzymes involved in starch synthesis have been well identified in cereals, but, until now, the regulatory mechanism underlying starch synthesis has not been fully understood, especially regarding the regulators on starch synthetic genes. As the key regulators mediating gene expression, the working models of transcriptional factors perform complicated networks during starch biosynthesis (Figure 1), and mainly depend on (i) the activation of different environmental factors; (ii) interaction with various factors, including other TFs; (iii) mediation of endogenous hormones; (iv) regulation of multiple genes by one TF; and (v) regulation of single gene by multiple TFs, and so on.

The race between the increasing starch demand and the shrinking arable land has resulted in the urgency to further understand starch biosynthesis and its regulation, which is vital to produce a rational design of agronomic traits and more and better polymers with biotechnological approaches. Besides, improved cereal grain quality is also essential to consumers; however, the formation of seed quality is a highly complex process. These processes are largely adjusted with various regulators, which perform precision guidance on the cooperation of starch synthesis-related proteins and lipids. Therefore, it is insufficient for the selection of any single trait in crop breeding.

Currently, the main application of cereal starch is to feed the increasing human population as a vital food source, and thus, high yields of cereals or improvement of states of starch shortage are still the key breeding targets. However, the enzymes involved in starch synthesis have been well identified in cereals, but the efforts to improve starch synthesis have failed using the simplistic one-enzyme strategy [98]. This was because precise cooperation of many modules was required in the process of starch synthesis, including enzymes, transporters, and regulators, i.e., TFs. Therefore, exploring more TFs and cis-elements is one potential strategy to mediate starch synthesis, especially for cells or tissues that lack starch [98,99]. However, compared with the abundant regulators that have been well identified in *Arabidopsis*, only dozens of regulators, especially for TFs, have been reported in cereal crops. Thus, the traditional mutagenesis technology, combined with advanced gene editing technology (i.e., CRISPR-Cas9), is a commonly used way to explore novel TFs, whose binding motifs can be determined with DAP-seq [100] and/or Chip-seq [101] technologies.

Besides, the building of regulatory networks can also help to design a rational strategy to improve starch biosynthesis. In order to achieve this, the selection and visualization of homogenized cells is key to the transcriptomic technologies [98] with the ancillary applications of fluorescent microscopy [102]. Recently, the successful application of single-cell RNA sequencing (scRNA-seq) in plants [103] provides another selective method to clarify the expression patterns of genes involved in starch synthesis, although it is technically challenging and expensive. Moreover, although still being controversial in legislations and ethics, the overexpression of genes via transgenic plants could serve as an effective and efficient way to improve starch synthesis, including the heterologous expression of genes derived from other cereals [71] and *Homo sapiens* [104]. The heterologous expression of wheat *SUSIBA2* in rice [71] produced a rice variety with high starch but low methane emission. Interestingly, recently, the successful introduction of *FTO*, one specific *Homo sapiens* gene associated with obesity and fat mass, into rice and potato largely increased the productivity and biomass [104]. Since FTO naturally exists in the human genome [104], it is feasible to introduce similar genes into cereals to improve starch synthesis.

Collectively, this review summarized the regulators underlying starch synthesis in cereal crops, and will help to provide potential applications in crop breeding and engineering efforts.

## Figures and Tables

**Table 1:** Transcriptional factors directly regulating starch biosynthesis in cereal crops.

TF	Types	Binding Domains	Target Genes	Species	Specific Expressed Tissues	References
REB/bZIP33	bZIP	ACGT	*GBSSI* (*Wx*) and *BEI*	Rice		[32]
RISBZ1/bZIP58	bZIP	ACGT	*AGPL3*, *GBSSI* (*Wx*), *SSIIa*, *BEI*, *BEIIb*, and *ISA2*	Rice	Endosperm	[11,28,29]
OsbZIP20	bZIP	ACGT	*GBSSI* (*Wx*)	Rice	Endosperm	[24]
ZmbZIP91	bZIP	ACTCAT	*AGPS1*, *SSI*, *SSIIIa*, and *ISA1*	Maize		[33]
TabZIP	bZIP		*GBSSI* (*Wx*) and *BEII*	Wheat	Endosperm	[26]
TabZIP28	bZIP	CACGTC	*AGPase*	Wheat	Endosperm	[27]
O2	bZIP		*SSIII*	Maize		[10]
OsETR2	AP2/ERF		*RAmy3D*	Rice	Endosperm	[34]
OsSERF	AP2/ERF		*RPBF*, *GBSSI*	Rice	Endosperm	[35]
OsBP-5	MYC-like	CAACGTG	*GBSSI* (*Wx*)	Rice	Endosperm	[12]
OsEBP-89	AP2/ERF	GCCAAC	*GBSSI* (*Wx*)	Rice	Endosperm	[12]
HvVP1/ABI3	AP2/ERF		*Amy6.4*	Barley		[36]
ZmABI4	AP2/ERF	ACCCG	*SSI*	Maize	Endosperm	[37]
ZmEREB156	AP2/ERF		*SSIIIa*	Maize	Endosperm	[38]
ZmEREB94	AP2/ERF		*SSI*	Maize		[39]
ZmNAC36	NAC		*AGPL2*, *AGPS2*, *SSI*, *GBSSIIb*, and *ISO2*	Maize	Endosperm	[15]
ZmNAC128	NAC	ACGCAA	*BT2*, *Zpu1*, GBSSI, *Sh2*, *SSV*, *ISA2*, and *SSIIa*	Maize	Endosperm	[40]
ZmNAC130	NAC	ACGCAA	*BT2*, *Zpu1*, GBSSI, *Sh2*, *SSV*, *ISA2*, and *SSIIa*	Maize	Endosperm	[40]
TaNAC019	NAC		*SSIIa*, *SuSy1*	Wheat	Endosperm	[41]
ZmMYB14	MYB		*BT1*	Maize	Endosperm	[16]
SUSIBA2	WRKY	SURE	*BEII*, *ISO1*	Barley	Endosperm	[42]
RPBF	DOF		*GBSSI*	Rice	Endosperm	[5]
RPBF	DOF	TGTAAAG	*RAmy1A*	Rice	Endosperm	[35]
ZmDOF3	DOF	AAAG	*SSIIa* and *SSIII*	Maize	Endosperm	[16]
ZmDOF36	DOF	DOF core elements	*GBSSI*, *ISA1*	Maize	Endosperm	[43]
BPBF	DOF	TGTAAAG	*α-amylase*	Barley		[19,44]
WPBF	DOF	TGTAAAG	*α-amylase*	Wheat		[45]

**Table 2:** Regulators indirectly affecting starch biosynthesis of cereal crops.

TF	Binding Domains	Target Genes	Specific Expressed Tissues	References
***Oryza sativa* L.**				
RISBZ1/bZIP58	TCCACGT(a/c)R(a/t) and GATGYRTGG	*O2*	Endosperm	[28]
***Zea mays* L.**				
ZmbZIP22	ACAGCTCA	*27-kDa γ-zein*	Endosperm	[86]
O2	GA/TGAPyPuTGPu	*PPDK*		[9,22,23]
O2	TCCACGTAGA	*22 kDa zein*	All types of tissues	[9,22,23]
OHP1	O2-likebox (TTTACGT)	*27-kDa γ-zein* and *22-kDa α-zein*		[87]
OHP2	O2-likebox (TTTACGT)	*zein*		[87]
ZmABI19	RY	*O2*, *PBF1*, *ZmbZIP22*, *NAC130*, *O11*	Endosperm and embryo	[88]
ZmNAC128	ACGCAA	*16-kDa γ-zein*	Endosperm	[40]
ZmNAC130	ACGCAA	*16-kDa γ-zein*	Endosperm	[40]
ZmMADS47	CATGT	*α-zein and 50-kDa γ-zein*	Endosperm	[45]
PBF/DOF13	TGTAAAG	*27-kD γ-* and *22-kD α-zein*	Endosperm	[10]
DOF1		*γ-zein*	Endosperm	[64]
***Triticum aestivum* L.**				
TaNAC019		*SPA*, *GaMyb*, *Glutenin*	Endosperm	[41]

## Data Availability

All data generated or analyzed during this study are included in this published article.
